# Systems pharmacology and transcriptomics reveal the mechanisms of Sanhuang decoction enema in the treatment of ulcerative colitis with additional *Candida albicans* infection

**DOI:** 10.1186/s13020-021-00487-2

**Published:** 2021-08-10

**Authors:** Zhijun Han, Xiaofen Tan, Juan Sun, Tianming Wang, Guiming Yan, Changzhong Wang, Kelong Ma

**Affiliations:** 1 College of Integrated Chinese and Western Medicine, College of Life Science, Anhui University of Chinese Medicine, Hefei, 230012 China; 2 Institute of Integrated Chinese and Western Medicine, Anhui Academy of Chinese Medicine, Hefei, 230012 China; 3 Anhui Provincial Key Laboratory of New Manufacturing Technology for Chinese Medicinal Decoction Pieces, Anhui University of Chinese Medicine, Hefei, 230012 China; 4 Key Laboratory of Xin’An Medicine, Ministry of Education, Anhui Academy of Chinese Medicine, Hefei, 230012 China

**Keywords:** Ulcerative colitis, *Candida albicans*, Sanhuang decoction, Systems pharmacology, Transcriptomics

## Abstract

**Background:**

Ulcerative colitis (UC) is an important inflammatory phenotype in bowel disease (IBD), which is caused by multiple potential factors, including fungal dysbiosis. *Candida albicans* (*C. albicans*) was confirmed to be an important factor promoting the occurrence and development of UC. Sanhuang decoction (SHD) has been used for UC therapy in China for thousand of years, although its core active constituents and pharmacological mechanism remain undefined.

**Methods:**

In this work, a murine model of UC with *C. albicans* colonization was established with dextran sodium sulfate (DSS) and *C. albicans* intragastric administration. The major bioactive constituents and potential mechanism of SHD against UC with fungal dysbiosis were comprehensively examined by combining systems pharmacology and in vivo transcriptomics.

**Results:**

SHD attenuated *C. albican*s burden, reduced DAI, increased mucosal integrity and relived systemic inflammation in UC mice. Systems pharmacology analysis identified 9 core bioactive ingredients and 45 hub targets of SHD against UC. Transcriptomics analysis confirmed 370 differentially expressed genes (DEGs) after SHD treatment, which were mainly enriched in inflammatory and immune response related signaling pathways. Toll-like receptor and PI3K-Akt signaling pathway were screened out as the candidate targets involved in the action of SHD on fungal dysbiosis-associated UC, which were consistent with the findings in systems pharmacology. The expression of TLR4, IL-1*β*, NF-κB, PI3K and Akt proteins were stimulated by *C. albican*s, and partially reversed by SHD in UC mice.

**Conclusion:**

These findings suggested SHD could be a candidate for the treatment of fungal dysbiosis-associated UC via TLR4-NF-κB and PI3K-Akt signaling pathways.

**Supplementary Information:**

The online version contains supplementary material available at 10.1186/s13020-021-00487-2.

## Background

Ulcerative colitis (UC) represents an important chronic inflammatory disease of the gastrointestinal (GI) tract featuring abdominal pain, diarrhea and rectal bleeding, with a significant impact on the quality of life [1]. Recently, the incidence and prevalence of UC have significantly increased in underdeveloped regions such as Asia and Africa, especially in China, where it was previously thought to be uncommon [2–4]. The pathogenesis of UC is complex, and several factors including genetics, environmental stimuli, immune disorder and the gut microbiota are considered to be involved in the occurrence and development of UC.

Fungi have long been suspected in UC pathogenesis, although they account for only 0.1% of the whole gut microbiota in healthy individuals [5]. Currently, growing evidence reveals that fungi are critical in maintaining intestinal homeostasis and the balance of immune response [6]. Several lines of evidence suggest that intestinal inflammation induces fungal proliferation [7]; on the other hand, certain fungal organisms modulate susceptibility to inflammation [7, 8]. *C. albicans (Ca)*, one of the most common opportunistic fungi in the human GI tract, is thought to be associated with UC development. Several reports showed that UC patients have excessive *C. albicans* load in the GI tract, and antifungal drugs can alleviate UC [5, 9–11]. In addition, *C. albicans* administration aggravates dextran sulfate sodium-induced colitis in the mouse model, with higher inflammatory cytokine secretion levels and severer mucosal damage [12, 13].

San-Huang Decoction (SHD), also known as San-Huang-Xie-Xin-Tang, is a classical traditional Chinese herbal formula, firstly recorded in the Synopsis of Prescriptions of the Golden Chamber written 2000 years ago. SHD consists of *Coptidis Rhizoma* (CR, Huanglian in Chinese), *Scutellariae Radix* (SR, Huangqin in Chinese) and *Radix Rhei Et Rhizome* (RR, Dahuang in Chinese), is traditionally used to clear damp-heat, remove blood stasis and detoxification. Emerging evidence shows that SHD can prevent cell apoptosis [14], suppress viral replication [15] and alleviate inflammation [16]. In addition, active compounds from SHD, e.g., berberine [17–19], baicalin [20–22] and emodin [23], have functions of anti-UC and modification of the gut microbiota components such as *C. albicans*. Previous studies revealed that SHD is broadly utilized in China for treating UC due to its synergistic and reliable effects [24, 25]. However, the major constituents and detailed mechanism of SHD in the treatment of *C. albicans*-associated UC remain unclear and need to be fully elucidated.

In the present study, we aimed to explore the potential role and underlying mechanism of SHD enema in UC with *C. albicans* dysbiosis. Mice with DSS-induced UC and *C. albicans* colonization were examined. The therapeutic effects of SHD were assessed by determining fungal burden, weight loss, DAI, histological score and serum contents of inflammatory factors. Systems pharmacology and transcriptomics were utilized to elucidate the molecular mechanisms of SHD in the treatment of UC involving *C. albicans*-associated dysbiosis. Post SHD treatment, the fecal fungal burden was reduced, and mucosal damage and systemic inflammation were alleviated. The PI3K-Akt and Toll like receptor signaling pathways were identified as SHD targets in treating the disorder. These findings provide novel insights in the mechanisms of SHD in the treatment of *C. albicans*-associated UC.

## Methods

### Animals and strains

Sixty specific pathogen-free (SPF) Kunming mice (female, 6 ~ 8 weeks, 20 ± 5 g) provided by Anhui Medical University Experimental Animal Center (Hefei, China, license No. SCXK Anhui 20170001) were housed at 18 ~ 25 °C and 50 ~ 70% relative humidity, under a 12 h light–dark cycle. Experiments involving animals had approval from the Animal Ethics Committee of Anhui University of Chinese Medicine.

*C. albicans* SC5314 was kindly provided by Professor Yuanying Jiang (Naval Medical University, Shanghai, China). Routine isolation and culture were performed as previously described [6].

### SHD preparation and quality control

*Coptidis Rhizoma* (CR), *Scutellariae Radix* (SR) and *Radix Rhei Et Rhizome* (RR) were purchased from Anhui Hospital of Chinese Medicine (Hefei, China). SHD enema was prepared as previously reported [24, 25]. The drugs were mixed at a ratio of 1:1:1 and extracted with 2 L of distilled water for 30 min. Then, the extract was boiled for 40 min. The cooled aqueous solution underwent centrifugation at 5000×*g*, collecting the supernatant, which underwent filtration through a 0.45 μm sterile microporous membrane. The filtrate was concentrated to 1.25 g/mL for further use. According to the requirement standards of the Pharmacopoeia of the People's Republic of China (2020 edition), the contents of berberine, palmatine, baicalin, rhein and emodin in SHD were detected by liquid chromatography-mass spectrometry (LC–MS). The conditions were as follow: an Agilent C18 (2.1 mm × 100 mm, 1.8 μm) with solvent A: 0.1% formic acid and solvent B: gradient methanol elution was used in the LC–MS detection system (1290–6460, Agilent, USA). The solvent flow rate was 0.3 mL/min in a column, at 40 °C. Berberine, palmatine and baicalin were performed on ESI + ionization modes, rhein and emodin were on ESI − ionization modes with data acquisition range from 10 to 500 Da. The measurements were done in triplicate samples, and the results were listed in Additional file 1: Fig. S1 and Additional file 2: Table S1.

### Animal model establishment and drug treatment

The animal model was established according to previous studies [6, 9]. In brief, mice were orally administrated 3.0% (w/v) dextran sulfate sodium (DSS, 36–50 kDa, MP Biomedicals) for consecutive 7 days. The animals with bloody or loose feces, with or without > 3 g body weight reduction at any time point were considered to have colitis. Then, the animals were randomized into 5 groups of 10 each, including the DSS, Model (DSS + Ca), SHD low dosage (SHD-L, 3.75 g/kg), SHD high dosage (SHD-H, 15.00 g/kg) and sulphasalazine (SASP, 0.72 g/kg) groups. In the Model group, mice were intragastrically administrated 1.0 × 10^8^ live *C. albicans* SC5314 for 4 days. In the SHD low and high groups, mice were subsequently treated with 3.75 g/kg and 15.00 g/kg SHD enema once daily for 7 days, respectively. Mice in the SASP group were given 0.72 g/kg SASP. Mice in the Control and Model groups were administered the same amount of saline. The administration of enema was performed as follows: mouse was fixed and faced upward to expose the anus. A enema syringe attached with a polyethylene catheter (outer diameter 2 mm) was inserted about 4 cm into the mouse rectum, the anus was squeezed, and 0.3 mL enema solution was slowly injected into the rectum for 30 s to 1 min, then the mouse was held upside down for 1 min to retain the enema solution. During this process, the enema solution was maintained at 37 °C, and all the instruments were treated aseptically. After mice were anesthetized with pentobarbital sodium (50 mg/kg, intraperitoneally), blood samples were collected by removing the left eyeball of the mice, and serum samples were kept at – 20 °C. Mice were sacrificed by cervical dislocation. Colons were excised and measured for length. Then, colon specimens were fixed with formalin or stored at -80℃ until use. The disease activity index (DAI) of each mouse was calculated daily, consisting of body weight loss (0, no loss; 1, 0–5%; 2, 5–10%; 3, 10–20%; 4, > 20%), stool consistency (0, normal; 2, loose stool; 4, diarrhea), and fecal blood (0, normal; 2, hemoccult; 4, gross bleeding) [26].

### *Candida albicans* burden assessment

*Candida albicans* amounts in the GIT were examined by plate counting after culturing fecal samples obtained from every mouse as described in our previous study [6]. Fecal specimens were weighed, resuspended in PBS and plated on Sabouraud dextrose agar supplemented with Komaga (No. K08A, Bio-engineering, Shanghai, China) for 48 h of culture at 37 °C. Yeast colonies were then counted, and expressed as CFU/10 mg feces.

### Detection of ASCA and β-glucan

Mouse serum samples were obtained, and the contents of ASCA (CK-E22252, Ruixin Biotech., Fujian, China) and β-glucan (F30161-B, Kexing Biotech., Shanghai, China) were assessed with ELISA kits as directed by the manufacturer.

### Histopathological analysis

Colon tissues were dissected and fixed with formalin, dehydrated, paraffin embedded and sectioned at 4 μm for hematoxylin and eosin (H&E) staining. Imaging and analysis were performed with a BX51 microscope (Olympus, Japan). The histopathological score was determined according to a previously published method [13].

### Serum TNF-α, IL-1β, IL-6 and IL-10 level evaluation

Serum was isolated by centrifugation of blood samples at 5000 rpm for 10 min. ELISA kits (MLBIO Biotechnology Co. Ltd. Shanghai, China) were utilized for determining the serum contents of TNF-*α* (ml002095), IL-1*β* (ml063132), IL-6 (ml002283) and IL-10 (ml002294).

### Systems pharmacology analysis

#### Screening of chemical components and prediction of related targets in SHD

The chemical ingredients of SHD were screened from the TCMSP (http://www.tcmspw.com/tcmsp.php) and TCMID (http://www.megabionet.org/tcmid/) databases. Ingredients meeting the demands of oral bioavailability (OB) ≥ 30% and drug-like property (DL) ≥ 0.18 were selected for identifying effective constituents. The potential targets of these compounds were screened in SwissTargetPrediction (http://www.swisstargetprediction.ch) based on SMILES strings which were obtained from PubChem (https://pubchem.ncbi.nlm.nih.gov/). The official name of each target gene was standardized based on the UniProtKB database (https://www.uniprot.org/).

#### Identification of ulcerative colitis-related targets

Ulcerative colitis related genes were searched and collected using the DisGeNET (http://www.disgenet.org) and GeneCards (https://www.genecards.org) databases with the keyword “ulcerative colitis, UC”. Repeated genes were discarded.

#### Protein–protein interactions and compound-target network building

Potential SHD’s therapeutic targets in UC were molecules simultaneously targeted by SHD constituents and UC. The STRING database (https://string-db.org/) was utilized for building a protein–protein interaction (PPI) network for these common targets. Cytoscape 3.7.1 (https://www.cytoscape.org/) was used to analyze the generated network. The final major targets of SHD in UC were selected based on Degree Centrality (DC), Betweenness Centrality (BC) and Closeness Centrality (CC), which are the mainly three categories of centrality in network analysis, accroding to network pharmacology evaluation method guidance [27]. Cytoscape was utilized to build a network of these main targets.

#### Gene ontology (GO) and Kyoto Encyclopedia of Genes and Genomes (KEGG) pathway enrichment analyses

GO and KEGG pathway enrichment analyses of key targets utilized the DAVID system (https://david.ncifcrf.gov/, v6.8) and R package. *P* < 0.05 was considered statistically significant.

### Transcriptomic analysis

Total RNA from colonic tissue samples in the Control, Model and SHD-H groups was isolated, enriched and purified with oligo-dT magnetic beads. Then, cDNA was synthesized, primed with poly-A and connected with sequencing adapters. Next, cDNA library construction and RNA sequencing were performed by Beijing Genomics Institution (Shenzheng, China). Clean reads were obtained with the SOAP nuke software by removing low quality reads from raw reads, and mapped to the reference genome (GCF_000001635.26_GRCm38.p6) by Bowtie2. Differentially expressed genes (DEGs) were obtained by the DEseq2 method according to the negative binomial distribution principle. The screening criteria for DEGs were FDR (false discovery rate) < 0.05 and log2|FC |≥ 2.

### Western blot

Total protein from colonic tissue specimens was isolated and resolved by SDS-PAGE. After transfer onto PVDF membranes, 5% skimmed milk was utilized for blocking. The blocked membranes underwent incubation with anti-TLR4 (AF7017, 1:1000), anti-NF-κB (AF5006, 1:1000), anti-p-NF-κB (AF2006, 1:1000), anti-PI3K (AF6241, 1:1000), anti-p-PI3K (AF3241, 1:1000), anti-Akt (AF6261, 1:500), anti-p-Akt (AF0016, 1:500), anti-IL-1*β* (AF5103, 1:1000) and anti-β-actin (AF5332, 1:1000) primary antibodies from Affinity Bioscience (Cincinnati, OH, USA) overnight, respectively. Then, the membranes were incubated with horseradish peroxidase-linked secondary antibodies (E-AB-1003; Elabscience, Wuhan, China). Signals were detected with an ECL imaging system (LAS4000, GE, Pittsburgh, PA, USA). ImageJ (National Institutes of Health, Bethesda, Maryland, USA) was used for quantitation.

### Immunohistochemistry

Colonic tissue sections underwent deparaffinization, rehydration and washing with PBS. Upon blocking with 10% goat serum, the sections underwent successive incubations with primary (overnight, 4 °C) and secondary (ambient, 1 h) antibodies. Then, diaminobenzidine was added as well as hematoxylin for counterstaining. A BX51 microscope was utilized for imaging at 200×.

### Statistical analysis

Data are mean ± SD, and were compared by one-way analysis of variance (ANOVA) with post hoc Tukey’s or student’s *t*-test. SPSS 22.0 (SPSS, Chicago, IL, USA) was used for data analysis. *P* < 0.05 indicated statistical significance.

## Results

### SHD exhibits therapeutic effects in mice with DSS colitis administered *C. albicans*

**Fig. 1:**
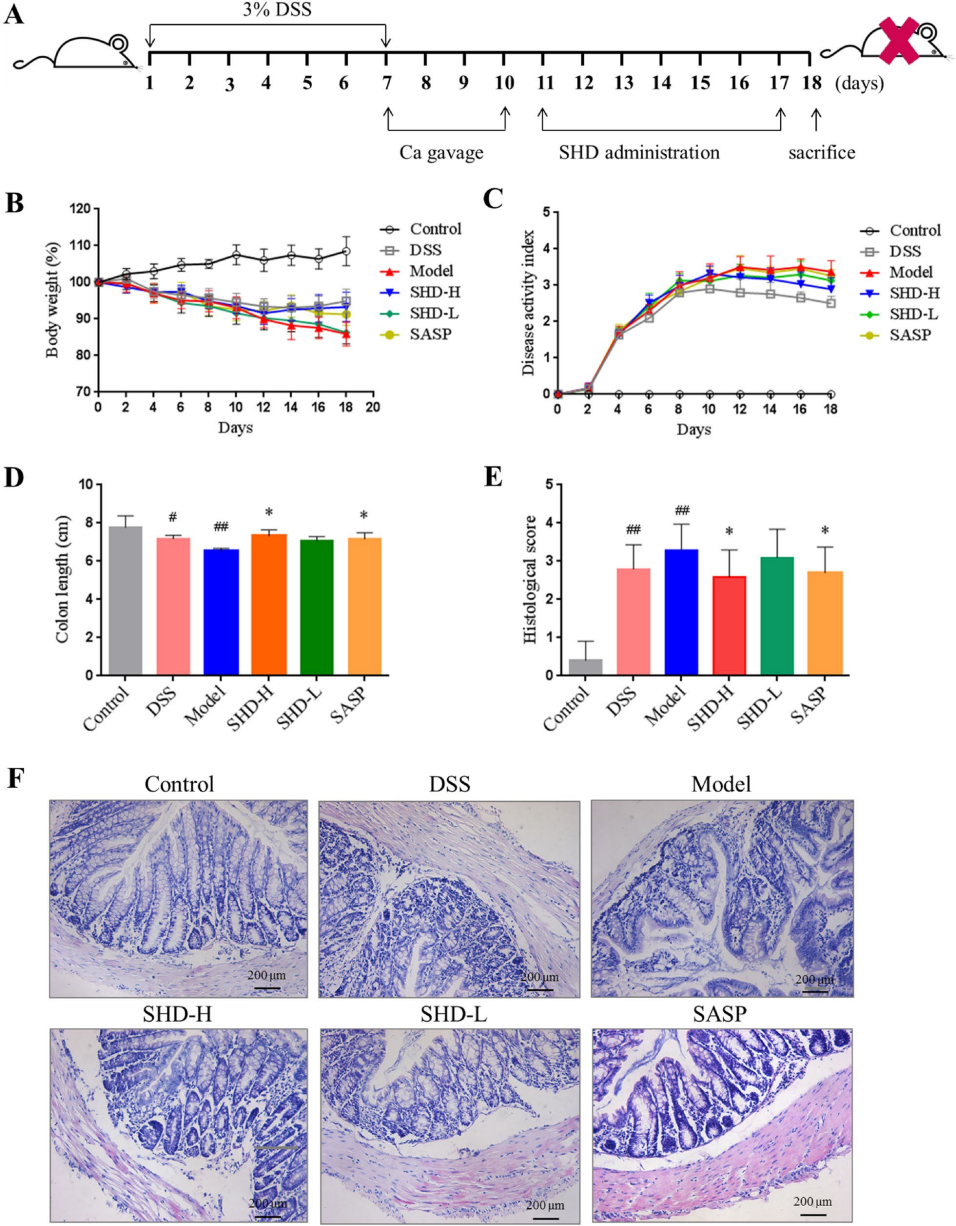
SHD attenuates the development of DSS colitis with *C. albicans. ***A** Experimental design of *C. albicans* administration in mice with colitis and SHD treatment. **B** Body weights. **C** Disease active index values. **D** Colon lengths. **E** Histological scores. **F** Colonic tissue samples after H&E staining (×200). Data are mean ± SD. ^#^*P* < 0.05, ^##^*P* < 0.01 versus normal group; *P < 0.05, **P < 0.01 versus Model group

A *C. albicans* colonized-UC animal model was established and described in Fig. 1A. *C. albicans* overtly aggravated UC as indicated by more pronounced body weight loss, elevated DAI and histological score, reduced colon length and more severe colonic epithelial mucosa damage compared with the DSS group (Fig. 1B–F, Additional file 1: Figure S2). These changes were mitigated after SHD administration. Compared with the Model group, body weight loss was reduced and DAI was decreased in the SHD groups (Fig. 1B, C). Besides, colon lengths were increased while histological scores were lower in the SHD-H group compared with the Model group (both *P* < 0.05) (Fig. 1D, E), suggesting that SHD-H had better effects than SHD-L.

### SHD reduces fungal burden and systemic inflammation in UC mice

**Fig. 2:**
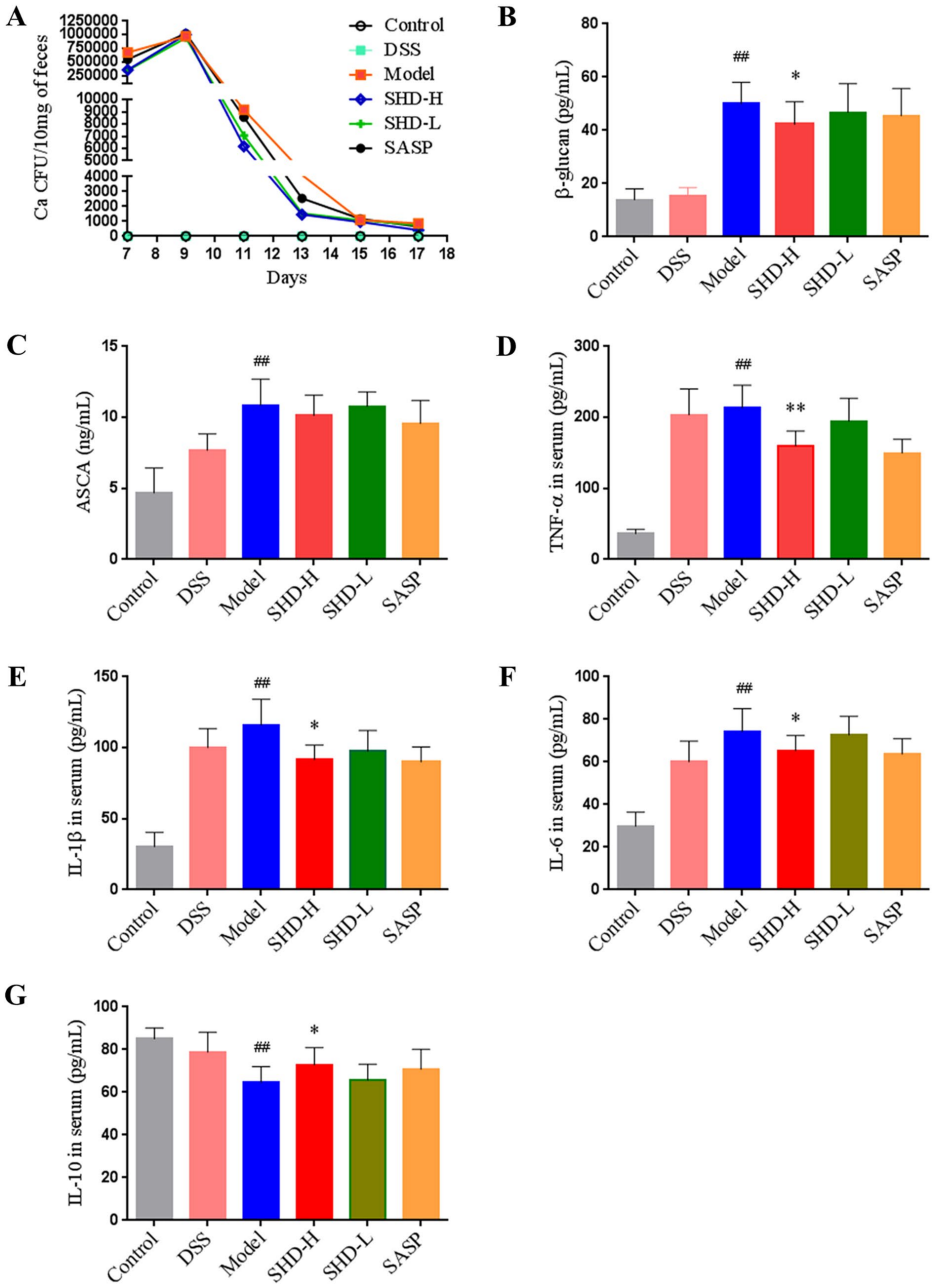
SHD decreases *C. albicans* burden and alleviates systemic inflammation in UC mice. **A** *C. albicans* load in feces was examined by agar plating at days 7, 9, 11, 13, 15 and 17, respectively. **B** Serum *β*-glucan and **C** anti-Saccharomyces cerevisiae antibodies (ASCA) amounts were assessed by ELISA. **D** Serum TNF-α, **E** IL-1*β*, **F** IL-6 and **G** IL-10 amounts were assessed by ELISA. Data are mean ± SD. ^#^*P* < 0.05, ^##^*P* < 0.01 versus normal group; *P < 0.05, **P < 0.01 versus Model group

*C. albicans* is not a commensal fungi in mice (36), and the mouse model of DSS colitis with *C. albicans* infection may highly mimic the human condition (additional *C. albicans*). Indeed, there was no *C. albicans* isolated from fecal samples in the Control and DSS alone groups. However, in the Model group, fecal *C. albicans* quantities were above 5 × 10^5^ CFU/10 mg at day 11 after gavage with *C. albicans* for 4 consecutive days (Fig. 2A). In addition, as shown in Fig. 2B, C, serum amounts of *β*-glucan (an important marker of fungal infection) and serum ASCA (an indicator of IBD) were increased in the Model group in comparison with the DSS and Control groups, implying aggravated systemic inflammation by *C. albicans* administration (*P* < 0.01). SHD had a certain anti-*C. albicans* effect in vivo*.* Compared with the Model group, the amounts *of C. albicans* were obviously decreased in the SHD-H group at days 12 and 13 (Fig. 2A). Moreover, serum *β*-glucan levels overtly decreased in the SHD-H group compared with the Model group (*P* < 0.05), while serum ASAC had no change among treatment groups.

Consistent with the aforementioned results, *C. albicans* inoculation worsened inflammation in mice with DSS colitis as indicated by higher serum amounts of TNF-*α*, IL-1*β* and IL-6 (proinflammatory cytokines) and lower levels of IL-10 (anti-inflammatory cytokine), compared with the DSS alone group (Fig. 2D–G). SHD effectively attenuated the systemic inflammation, as serum amounts of TNF-*α*, IL-1*β* and IL-6 were reduced while IL-10 levels were elevated in the SHD-H group in comparison with the Model group (*P* < 0.05 or 0.01) (Fig. 2D–G).

### Active components of SHD

To decipher the underlying mechanism of action of SHD in UC treatment, we screened potential active compounds using a well-established in silico ADME pipeline. We obtained 283 candidate compounds (48 CR, 143 SR and 92 RR) from the TCMSP database, and determined their corresponding ADME parameters, including OB and DL. Of these constituents, 66 (14 CR, 36 SR and 16 RR) passed the drug screening criteria (OB ≥ 30% and DL ≥ 0.18), and were retained for further processing. The potential active constituents and respective targets in SHD are listed in Table 1. Of these targets, quercetin had 154 targets, making it the compound with the most targets in SHD, followed by wogonin (45 targets) and beta-sitosterol (38 targets).

**Table 1:** The potential active compounds and targets in SHD

Drug	Molecule name	OB (%)	DL	Target number
CR1	berberine	36.86	0.78	17
CR	Obacunone	43.29	0.77	0
CR2	berberrubine	35.74	0.73	13
CR(B1)	epiberberine	43.09	0.78	11
CR3	(R)-Canadine	55.37	0.77	31
CR4	Berlambine	36.68	0.82	20
CR5	Corchoroside A_qt	104.95	0.78	2
CR6	Magnograndiolide	63.71	0.19	4
CR	Palmidin A	35.36	0.65	0
CR7	palmatine	64.6	0.65	19
CR8	quercetin	46.43	0.28	154
CR(B2)	coptisine	30.67	0.86	9
CR9	Worenine	45.83	0.87	7
CR	Moupinamide	86.71	0.26	0
SR1	acacetin	34.97	0.24	26
SR2	wogonin	30.68	0.23	45
SR3	(2R)-7-hydroxy-5-methoxy-2-phenylchroman-4-one	55.23	0.2	21
SR4	baicalein	33.52	0.21	37
SR	5,8,2ʹ-Trihydroxy-7-methoxyflavone	37.01	0.27	0
SR5	5,7,2,5-tetrahydroxy-8,6-dimethoxyflavone	33.82	0.45	13
SR6	Carthamidin	41.15	0.24	4
SR	2,6,2ʹ,4ʹ-tetrahydroxy-6ʹ-methoxychaleone	69.04	0.22	0
SR7	Dihydrobaicalin_qt	40.04	0.21	3
SR8	Eriodyctiol (flavanone)	41.35	0.24	8
SR9	Salvigenin	49.07	0.33	18
SR10	5,2ʹ,6ʹ-Trihydroxy-7,8-dimethoxyflavone	45.05	0.33	17
SR11	5,7,2ʹ,6ʹ-Tetrahydroxyflavone	37.01	0.24	6
SR	dihydrooroxylin A	38.72	0.23	0
SR12	Skullcapflavone II	69.51	0.44	21
SR13	oroxylin a	41.37	0.23	26
SR14	Panicolin	76.26	0.29	14
SR15	5,7,4ʹ-Trihydroxy-8-methoxyflavone	36.56	0.27	18
SR16	NEOBAICALEIN	104.34	0.44	22
SR17	DIHYDROOROXYLIN	66.06	0.23	11
SR(A)	beta-sitosterol	36.91	0.75	38
SR18	sitosterol	36.91	0.75	3
SR19	Norwogonin	39.4	0.21	12
SR20	5,2ʹ-Dihydroxy-6,7,8-trimethoxyflavone	31.71	0.35	21
SR21	ent-Epicatechin	48.96	0.24	6
SR22	Stigmasterol	43.83	0.76	31
SR(B2)	coptisine	30.67	0.86	9
SR23	bis[(2S)-2-ethylhexyl] benzene-1,2-dicarboxylate	43.59	0.35	1
SR	Supraene	33.55	0.42	0
SR24	Diop	43.59	0.39	3
SR(B1)	Epiberberine	43.09	0.78	11
SR25	Moslosooflavone	44.09	0.25	25
SR26	11,13-Eicosadienoic acid, methyl ester	39.28	0.23	1
SR27	5,7,4ʹ-trihydroxy-6-methoxyflavanone	36.63	0.27	6
SR28	5,7,4ʹ-trihydroxy-8-methoxyflavanone	74.24	0.26	6
SR29	rivularin	37.94	0.37	22
RR1	EUPATIN	50.8	0.41	16
RR	Mutatochrome	48.64	0.61	0
RR2	Physciondiglucoside	41.65	0.63	1
RR	Procyanidin B-5,3ʹ-O-gallate	31.99	0.32	0
RR3	rhein	47.07	0.28	7
RR	Sennoside E_qt	50.69	0.61	0
RR4	Torachrysone-8-O-beta-d-(6ʹ-oxayl)-glucoside	43.02	0.74	1
RR5	Toralactone	46.46	0.24	9
RR6	Emodin-1-O-beta-d-glucopyranoside	44.81	0.8	1
RR	Sennoside D_qt	61.06	0.61	0
RR7	Daucosterol_qt	35.89	0.7	2
RR	palmidin A	32.45	0.65	0
RR (A)	beta-sitosterol	36.91	0.75	38
RR8	aloe-emodin	83.38	0.24	24
RR	gallic acid-3-O-(6ʹ-O-galloyl)-glucoside	30.25	0.67	0
RR9	(−)-catechin	49.68	0.24	11

### Core compounds screening and hub target network construction

**Fig. 3:**
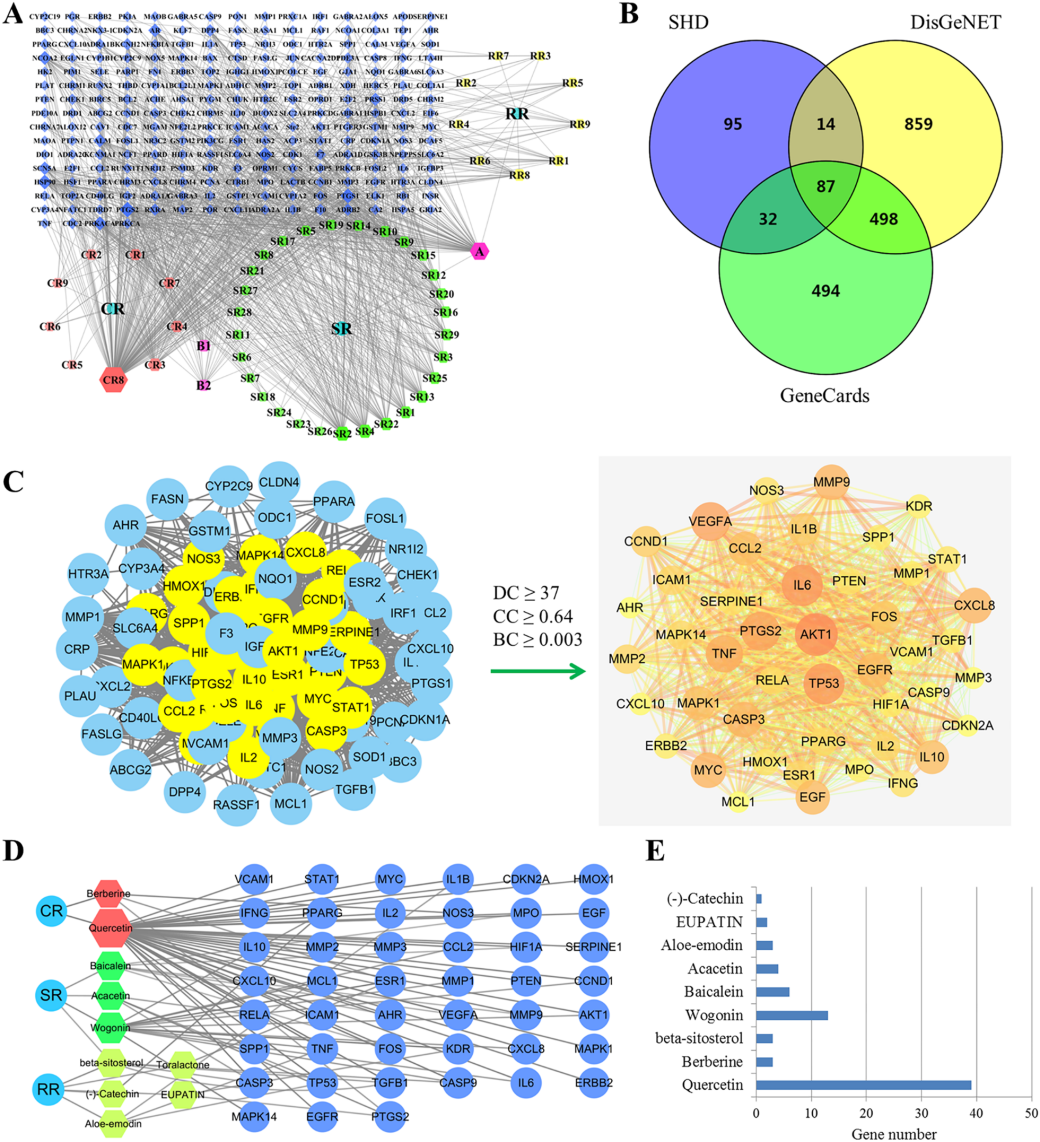
Core constituents and hub targets of SHD in UC. **A** Compound-Target network of SHD. Pink nodes represent CR, green nodes represent SR, yellow nodes represent RR, and blue nodes represent the targets. The purple node A represents a compound shared by SR and RR; the purple nodes B1 and B2 represent compounds shared by CR and SR. **B** Venn diagram of targets shared by SHD and UC. **C** The topological screening for the PPI network. **D** Network of core constituents and targets. **E** The numbers of targets for the 9 core substituents

Swiss Target Prediction and the TCMSP database were utilized to identify compound-related targets, and the compound-target network was built with Cytoscape. Post discarding the compounds with no predicted target, we obtained 11 active constituents with 182 predicted targets in CR, 32 active compounds with 122 targets in SR, and 10 active constituents with predicted 71 targets in RR, totaling 342 non-redundant targets in SHD (Fig. 3A, Table 1). The UC-related target genes were screened using GeneCards and DisGeNet databases, finally yielding 1984 UC-related targets. Then, the targets of SHD were matched with those of UC, and a total of 87 SHD-UC targets were determined (Fig. 3B).

To further explore the detailed functions of the 87 SHD-UC targets, a protein–protein interaction analysis was conducted with an online tool, the STRING database. A network comprising 87 targets was built, with 1602 edges (medium confidence score > 0.4). The core network, comprising 45 nodes and 838 edges, was generated based on DC above median value (37), BC ≥ median BC value (0.003) and CC value ≥ median CC (0.64) in topological network analysis (Fig. 3C). These 45 hub genes were mainly regulated by 9 core ingredients, i.e., quercetin and berberine from CR; wogonin, baicalein, acacetin and beta-sitosterol from SR; and aloe-emodin, EUPATIN and (−)-catechin from RR (Fig. 3D). Quercetin had the most targets (39 targets), followed by wogonin (13 targets) and baicalein (6 targets; Fig. 3E), suggesting their key roles in anti-UC function.

### GO and KEGG pathway enrichment analyses

**Fig. 4:**
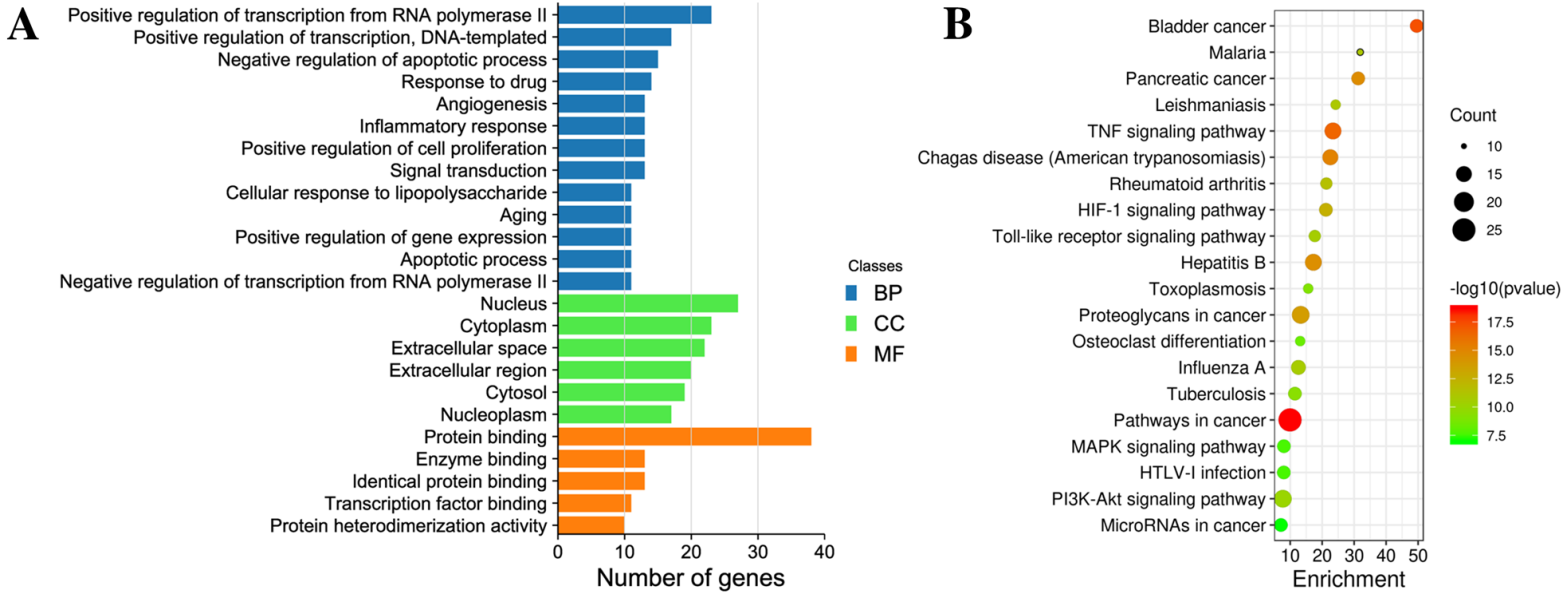
GO function and KEGG pathway analyses of the hub genes. **A** Top 15 biological processes (BP), 6 cellular components (CC) and 5 molecular functions. **B** Top 20 KEGG pathways

To investigate the detailed functions of the above 45 hub targets, GO function and KEGG pathway enrichment analyses were carried out. In GO analysis, these targets were primary enriched in cellular components of nucleus, cytoplasm and extracellular space, and mainly involved in protein binding, enzyme binding and tanscription factor binding as molecular functions (Fig. 4A). Regarding biological processes, these core targets were primary involved in positive regulation of transcription, negative regulation of apoptosis, response to drugs, angiogenesis and inflammatory response (Fig. 4A).

KEGG pathway analysis showed the hub genes highly participated in pathways related to cancer (25 genes), PI3K-Akt signaling (17 genes), TNF signaling (16 genes), HIF-1 signaling (13 genes), MAPK signaling (13 genes) and Toll-like receptor signaling (12 genes), indicating the underlying molecular mechanisms of SHD in UC treatment (Fig. 4B).

### Identification of differentially expressed genes post-SHD treatment in mice with *C. albicans*-associated colitis

**Fig. 5:**
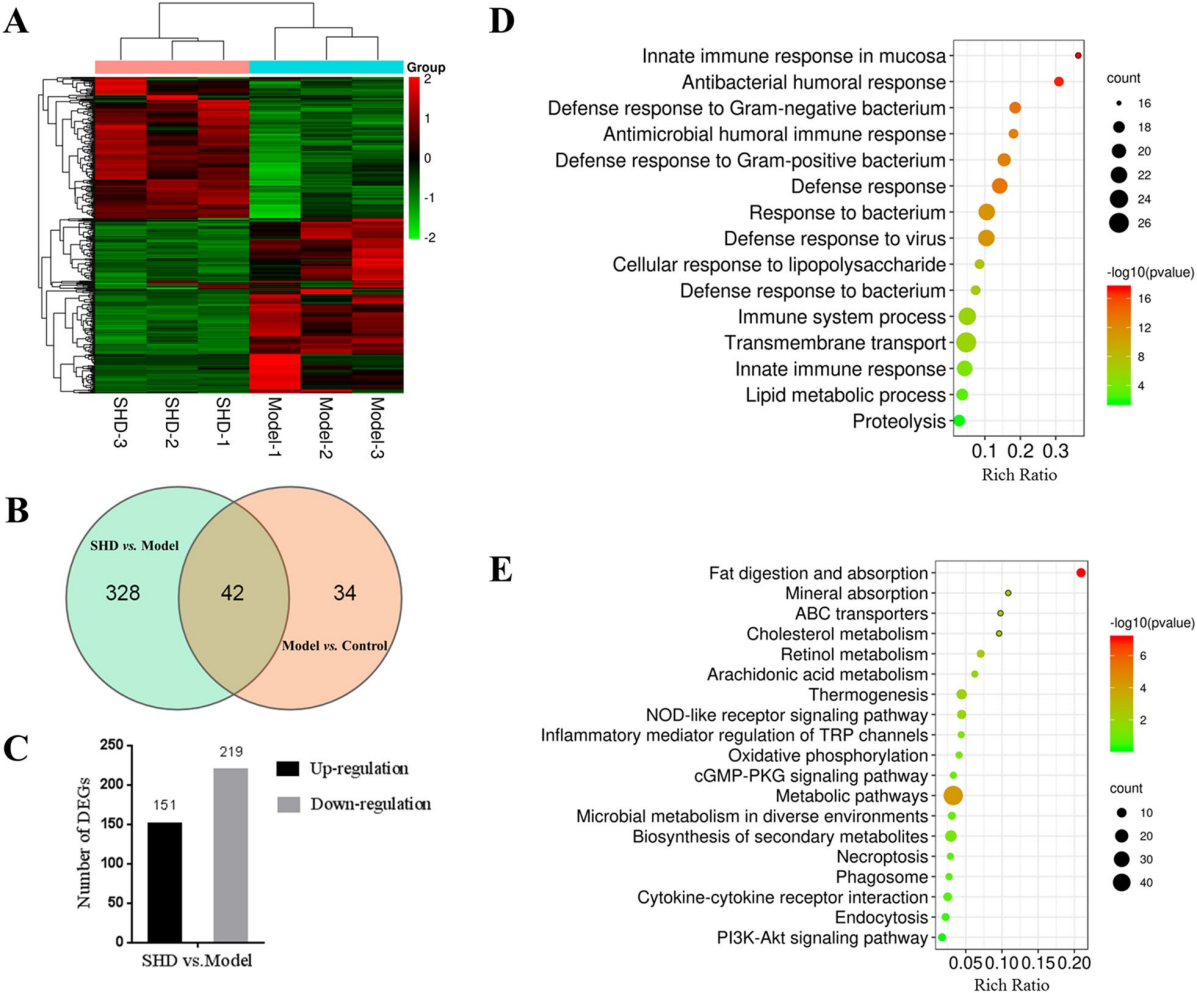
Transcriptomics analysis of SHD’s effects in *C. albicans*-associated colitis in mice. **A** Expression heat map of DEGs between the SHD and Model groups. **B** Venn diagram of targets shared in SHD group versus Model group, and Model group versus Control group. **C** Up- and down-regulated genes in SHD group versus Model group. **D** GO enrichment analysis of DEGs in SHD group versus Model group; the top 15 biological processes are shown. **E** KEGG pathway enrichment analysis of DEGs in SHD group versus Model group (*P* < 0.05)

Colonic tissue transcriptomics analysis was conducted for elucidating the functional genes affected by SHD. Totally 9 samples in three groups (i.e., Control, Model and SHD groups, three samples per group) were tested, and an average yield of 6.63G data per sample was obtained. Totally 18,240 genes were found, with 17,241 in the Control group, 17,624 in the Model group and 17,522 in the SHD group, with 16,722 genes shared among these three groups (Additional file 1: Figure S3). Compared with the Model group, 370 genes were differentially expressed (DEGs) in the SHD group (Fig. 5A, B), including 151 upregulated and 219 downregulated (Fig. 5C).

### Functions of DEGs

GO analysis revealed DEGs were primarily involved in transmembrane transport (26 genes), immune system process (23 genes), response to a bacterium (22 genes), innate immune response (21 genes) and innate immune response in mucosa (16 genes) (Fig. 5D, Additional file 1: Figure S4).

KEGG pathway analysis revealed DEGs were basically enriched in inflammatory and immune response-related signaling pathways, including PI3K-Akt signaling, NOD-like receptor signaling, Cytokine-cytokine receptor interaction, Inflammatory mediator regulation of TRP channels and Arachidonic acid metabolism (Fig. 5E). Significantly, genes enriched in the PI3K-Akt and NOD-like receptor pathways were downregulated in the colon tissue of SHD treated mice, indicating SHD may exert antifungal-UC effects through these targets and pathways.

### SHD attenuates *C. albicans*-associated colitis via the TLR4-NF-κB and PI3K-Akt pathways

**Fig. 6:**
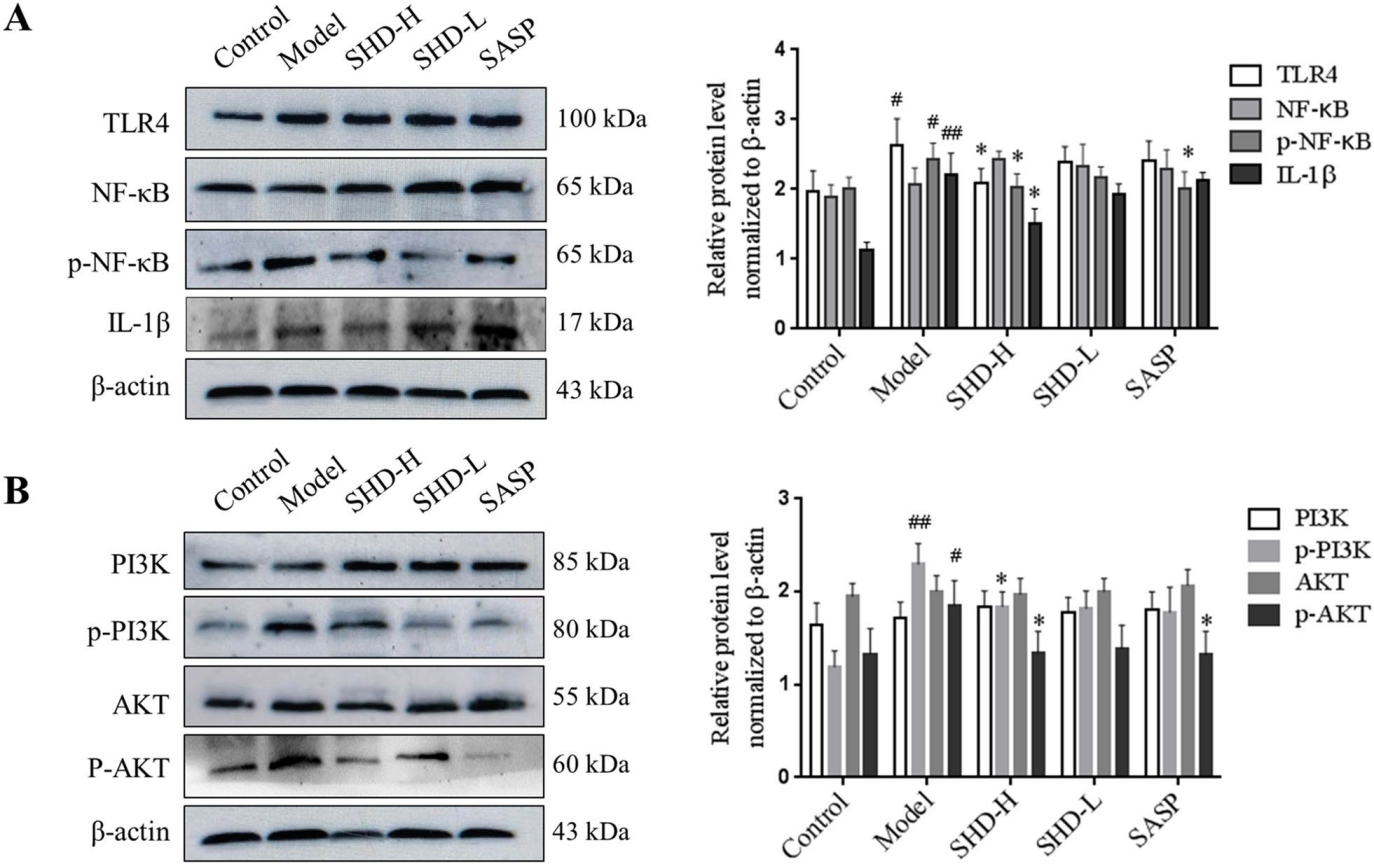
Protein expression levels in colon tissue samples examined by immunoblot. **A** TLR4, NF-κB, p-NF-κB and IL-1β. **B** PI3K, p-PI3K, AKT and p-AKT. Staining intensity was assessed with ImageJ. Data are mean ± SD, ^#^*P* < 0.05, ^##^*P* < 0.01 versus normal group; *P < 0.05, **P < 0.01 versus Model group

**Fig. 7:**
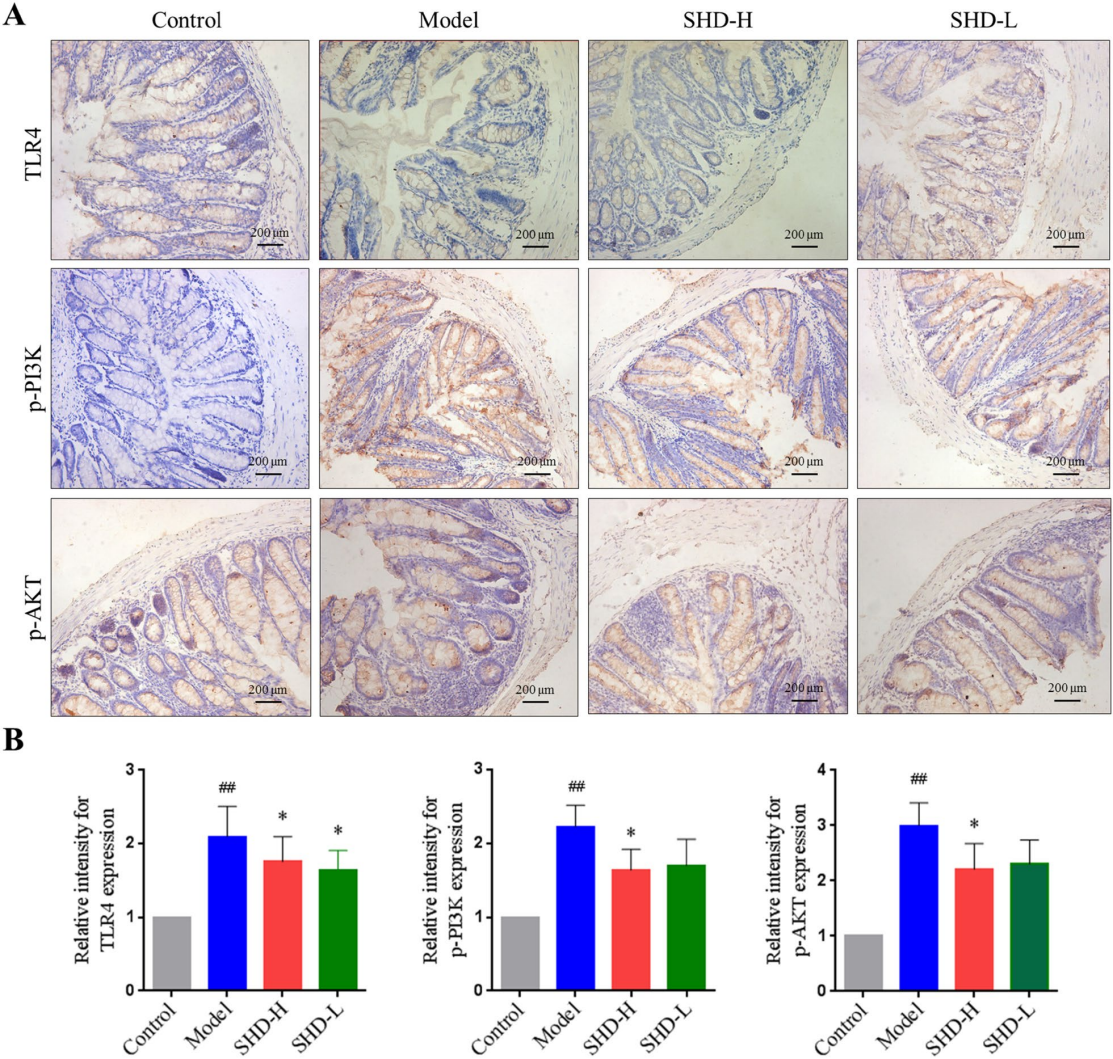
TLR4, p-PI3K and p-AKT amounts in colon tissue samples detected by immunohistochemistry. **A** Representative IHC images for TLR4, p-PI3K and p-AKT signals are shown (×200). **B** Staining intensity was examined with Image-Pro 6.0. Data are mean ± SD, ^#^*P* < 0.05, ^##^*P* < 0.01 versus normal group; *P < 0.05, **P < 0.01 versus model group

For further validating SHD targets in *C. albicans*-associated colitis, key TLR4-NF-κB and PI3K-Akt pathway effectors were examined. TLR4 and IL-1*β* amounts were overtly increased in the Model group in comparison with the Control group (*P* < 0.05; Figs. 6A, 7A, B). Although NF-κB, PI3K and Akt amounts had no differences between the Model and Control groups, p–NF-κB, p–PI3K and p–Akt levels were much higher than those of the Control group (*P* < 0.05 or 0.01; Figs. 6A, B, 7A, B). Aberrant expression of TLR4, IL-1*β*, p–NF-κB, p–PI3K and p–Akt was reversed by SHD, which was consistent with the above findings in systems pharmacology and transcriptomics (*P* < 0.05; Figs. 6A, B, 7A, B).

## Discussion

Recently, gut fungi dysbiosis has been recognized as an important factor involved in UC pathogenesis [5, 28]. As one of the predominant fungi related to IBD, *C. albicans* is not commensal in the mouse gut, which differs from humans; therefore, DSS induced colitis with *C. albicans* infection may more adequately mimic the human disease [12, 29]. In this study, we established this model as previously described, with minor modifications [9]. Briefly, a *C. albicans*-colonized UC model was established via exposure to 3.0% DSS in drinking water for 7 days, followed by administration of 1.0 × 10^8^ *C. albicans* live cells/mL by continuous gavage for 4 days. We found that *C. albicans* aggravated DSS-induced colitis, with higher DAI, histological score and serum levels of inflammatory factors versus DSS alone group. Therefore, this model could resemble the status of IBD with *C. albicans* overgrowth in humans.

Traditional Chinese medicine has been broadly applied for treating UC in China for a long time due to its safety and efficacy in UC. Several classical prescriptions, including Shaoyao decoction [30], Huangqing decoction [31] and Huang-Lian-Jie-Du decoction [32], were verified to exhibit therapeutic effects in UC. These effective formulas perform several common functions such as heat-clearing, dampness-drying and detoxification, which are also carried out by SHD. However, few studies have revealed the anti-UC and anti-inflammatory functions of SHD, and whether SHD enema is effective against UC with *C. albicans* colonization remains unknown. Here, we found that SHD could effectively ameliorate UC symptoms in this animal model. SHD administration decreased body weight loss, DAI score and colon length shortening. Moreover, SHD reduced local and systemic *C. albicans* burdens, inhibited mucosal and submucosal damage, and relived inflammatory response, which was consistent with previous study [24]. Taken together, SHD enema may be a potential strategy to overcome UC with *C. albicans* dysbiosis.

The complex composition and multiple action patterns of TCM make it hard to understand its detailed therapeutic mechanisms at the molecular level [33]. Network pharmacology, a new systems approach for exploring new active ingredients and potential action mechanisms for TCM preparations [34], has been widely used in pharmacological research of TCM against UC [35–38]. Using this method, 9 core ingredient and 45 related key targets were identified, and quercetin, wogonin and baicalein were found to be probably the key active compounds of SHD in UC therapy. GO and KEGG pathway analyses revealed these 45 hub genes were mostly involved in immune and inflammatory responses, with roles in the PI3K-Akt, TNF, HIF-1, MAPK and Toll-like receptor pathways.

Transcriptomics is a high-throughput technique to observe mRNAs with differential expression in different tissues and time points in organisms, playing a major role in the study of drug action mechanism [39, 40]. In this study, 370 differentially expressed genes, including 151 upregulated and 219 downregulated, were screened. Function enrichment results for these DEGs partly coincided with those of network pharmacology. GO function analysis revealed that these DEGs were primary involved in biological process, immune system process, innate immune response and innate immune response in mucosa. KEGG analysis showed these DEGs were basically enriched in PI3K-Akt and NOD-like receptor pathways. Taken together, these results indicate the relative accuracy of our network in predicting SHD targets, and support the reliability of transcriptome sequencing results.

The Toll-like receptor signaling pathway, an important signaling pathway that detects and removes pathogens, is involved in UC pathogenesis and targeted by many Chinese medicine preparations against ulcerative colitis [41–43]. The transcriptome data in this study suggested that Toll-like receptor signaling-related genes were downregulated after SHD treatment. Indeed, TLR4, NF-κB and IL-1*β* were downregulated in the SHD group in comparison with the Model group, indicating that SHD might play an anti-UC role by inhibiting the TLR4/ NF-κB signal pathway.

PI3K-Akt signaling represents another important pathway contributing to the pathogenesis of ulcerative colitis [44]. Huang et al. found that wortmannin (a PI3K inhibitor) reduces p-Akt and TNF-α amounts in colon tissue samples from DSS-treated mice, and significantly alleviates inflammation in colitis [45]. Some TCMs or natural compounds exert their anti-UC effects via PI3K-Akt signaling. For example, oxymatrine markedly ameliorates UC via anti-inflammatory and pro-apoptotic mechanisms, suppressing Th1 and Th17 cell differentiation through PI3K-AKT signaling [46]. Baicalin alleviates the severity of colon inflammation by blocking the TLR4/NF-κB-p65/IL-6 signaling pathway [47]. We found that PI3K and Akt phosphorylation levels in the SHD group were decreased significantly, suggesting that SHD may inhibit UC with *Candida albicans* colonization by suppressing the PI3K-AKT pathway.

## Conclusion

Taken together, network pharmacology and transcriptomics data suggested SHD could alleviate UC with *C. albicans* colonization via synergistic effects of many constituents and targets.

We propose Sanhuang decoction enema may function as an effective anti-UC approach by reducing the additional impact of *C. albicans* on the progression of DSS-induced colitis, decreasing fecal fungal burden and lowering systemic inflammation mainly via the TLR4/NF-kB and PI3K-Akt pathways. The present work uncovered the mechanism of SHD in UC with *C. albicans* dysbiosis and provided a scientific basis for a rational application of traditional Chinese medicine.

## Supplementary Information

**Additional file 1: Figure S1.** LC-MS chromatograms of main ingredients in SHD. **Figure S2.** Representative photographs of colon samples. **Figure S3.** Venn diagram of targets shared in the Control, Model and SHD groups. **Figure S4.** GO analysis of DEGs in SHD group versus Model group. (A) Top 15 molecular functions. (B) Top 14 cellular components.

**Additional file 2: Table S1.** The contents of main ingredients in SHD.

## Data Availability

The data set supporting the results of this article are included within the article and Additional files.

## Abbreviations

BC: Betweenness centrality
CC: Closeness centrality
CR: *Coptidis Rhizoma*
DAI: Disease activity index
DC: Degree centrality
DEGs: Differentially expressed genes
DL: Drug-like property
GI: Gastrointestinal
GO: Gene ontology
H&E: Hematoxylin and eosin
KEGG: Kyoto encyclopedia of genes and genomes
LC–MS: Liquid chromatography–mass spectrometry
OB: Oral bioavailability
PPI: Protein–protein interaction
RR: *Radix Rhei Et Rhizome*
SHD: San-Huang decoction
SPF: Specific pathogen-free
SR: *Scutellariae radix*
UC: Ulcerative colitis
