# Supplementary material for: Systems pharmacology and transcriptomics reveal the mechanisms of Sanhuang decoction enema in the treatment of ulcerative colitis with additional Candida albicans infection
Source: Chin Med. 2021 Aug 10;16:75. doi: 10.1186/s13020-021-00487-2 (PMC8353752; doi:10.1186/s13020-021-00487-2)
Supplement: Supplementary file 1 — Additional file 1: Figure S1. LC-MS chromatograms of main ingredients in SHD. Figure S2. Representative photographs of colon samples. Figure S3. Venn diagram of targets shared in the Control, Model and SHD groups. Figure S4. GO analysis of DEGs in SHD group versus Model group. (A) Top 15 molecular functions. (B) Top 14 cellular components. [file 13020_2021_487_MOESM1_ESM.docx]

**
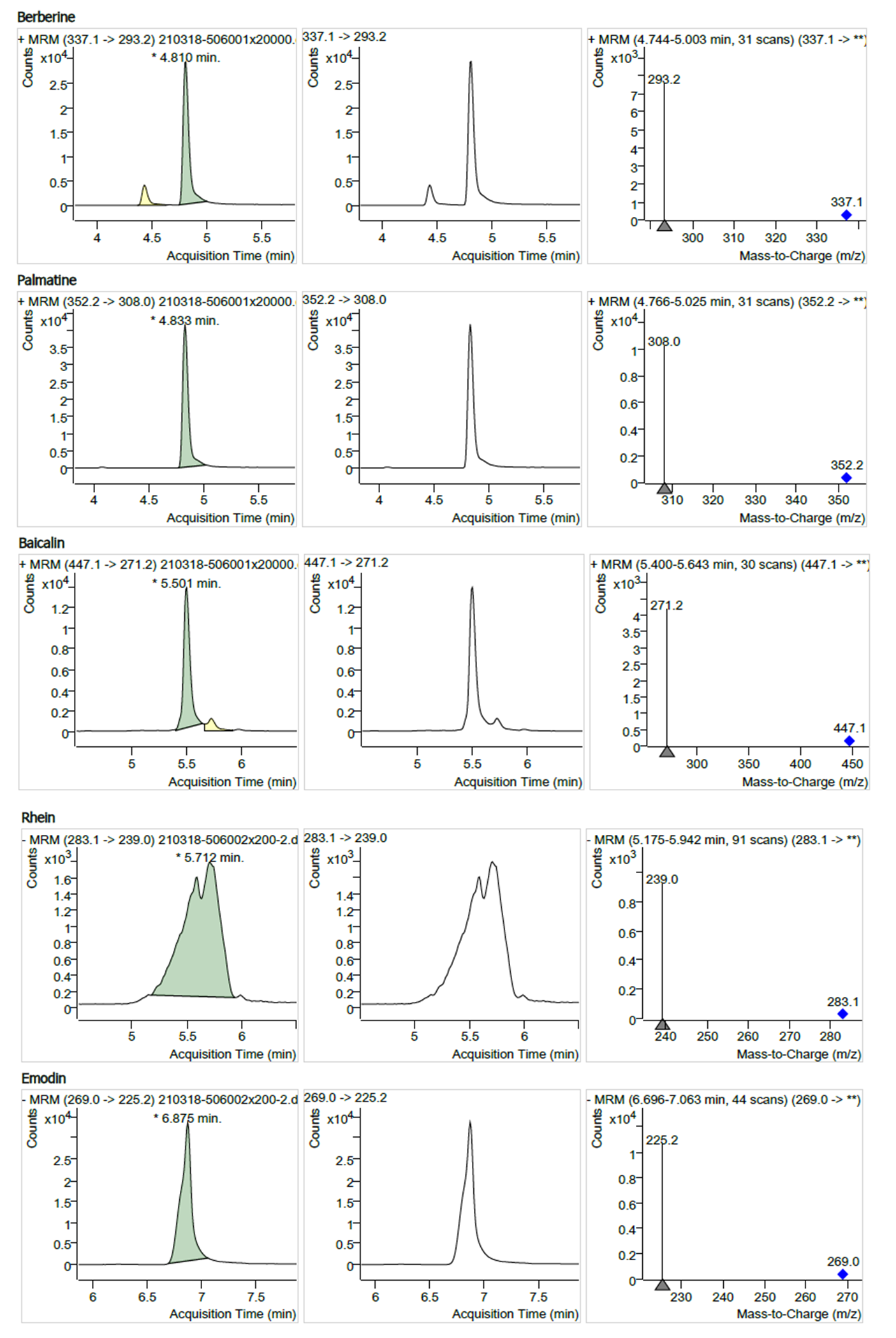
**

**Additional file Figure S1** LC-MS chromatograms of main ingredients in SHD

**
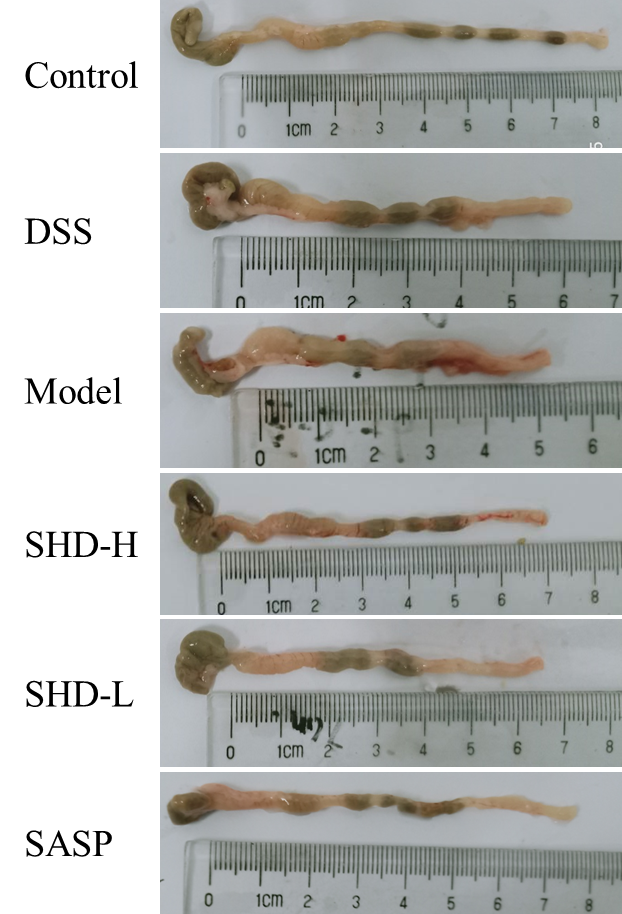
**

**Additional file Figure S2** Representative photographs of colon samples.

**
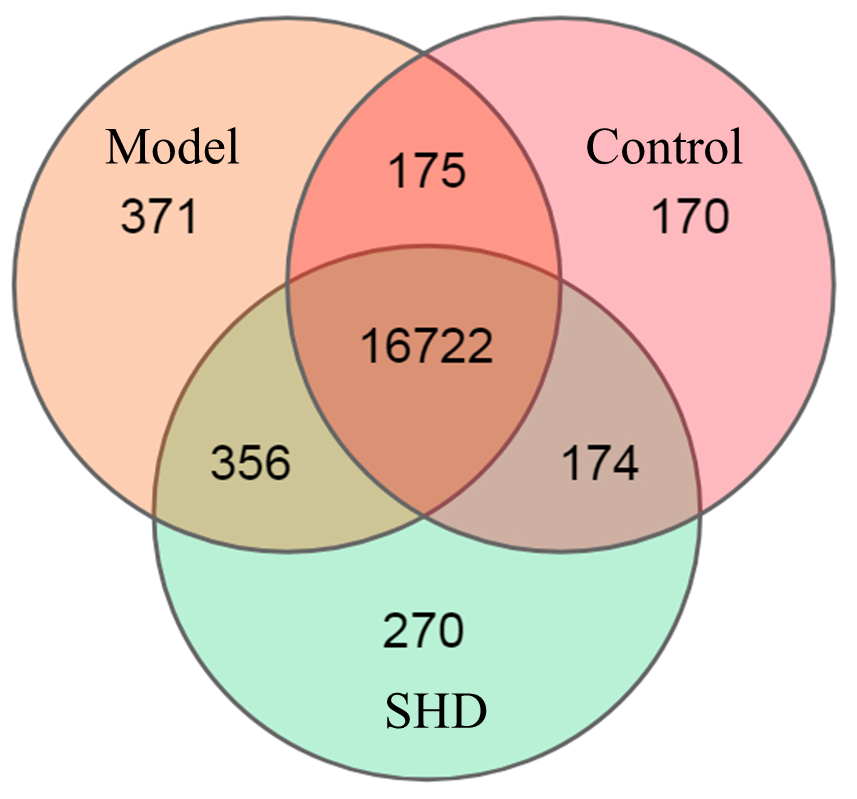
**

**Additional file Figure S3** Venn diagram of targets shared in the Control, Model and SHD groups.

**
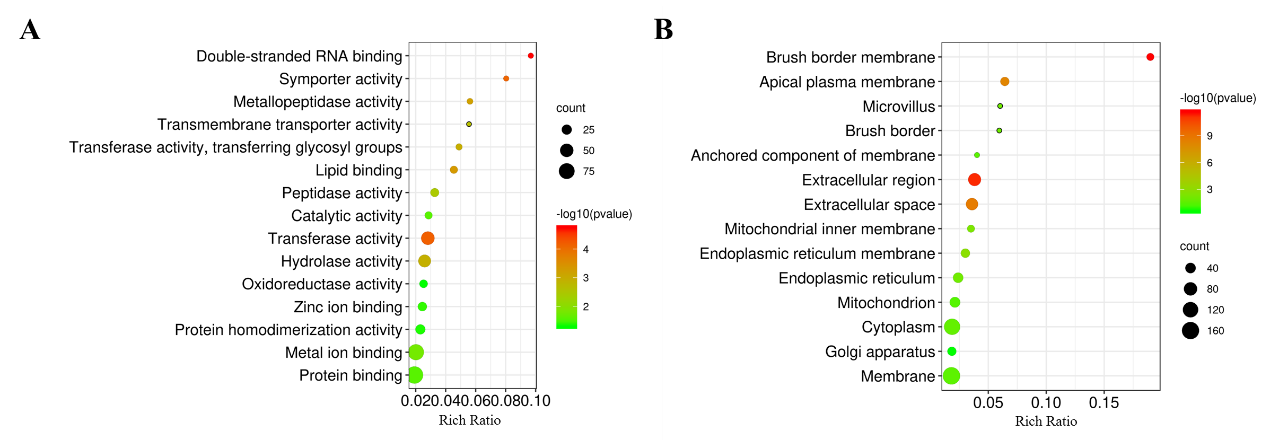
**

**Additional file Figure S4** GO analysis of DEGs in SHD group versus Model group. (A) Top 15 molecular functions. (B) Top 14 cellular components.
