# Supplementary material for: Systems pharmacology and transcriptomics reveal the mechanisms of Sanhuang decoction enema in the treatment of ulcerative colitis with additional Candida albicans infection
Source: Chin Med. 2021 Aug 10;16:75. doi: 10.1186/s13020-021-00487-2 (PMC8353752; doi:10.1186/s13020-021-00487-2)
Supplement: Supplementary file 2 — Additional file 2: Table S1. The contents of main ingredients in SHD. [file 13020_2021_487_MOESM2_ESM.docx]

Additional file Table S1 The contents of main ingredients in SHD

| Constituent | Sample 1  (μg/mL) | Sample 2  (μg/mL) | Sample 3  (μg/mL) | Mean value  (μg/mL) |
| --- | --- | --- | --- | --- |
| berberine | 0.6486 | 0.6508 | 0.6583 | 0.653 |
| palmatine | 0.2581 | 0.2604 | 0.2670 | 0.262 |
| baicalin | 3.7317 | 3.7432 | 3.7185 | 3.731 |
| rhein | 0.0113 | 0.0113 | 0.0111 | 0.011 |
| emodin | 0.0273 | 0.0274 | 0.0272 | 0.027 |
